# Patient centred care for the medical treatment of lower urinary tract symptoms in patients with benign prostatic obstruction: a key point to improve patients’ care – a systematic review

**DOI:** 10.1186/s12894-018-0376-x

**Published:** 2018-06-26

**Authors:** Cosimo De Nunzio, Fabrizio Presicce, Riccardo Lombardo, Alberto Trucchi, Mariangela Bellangino, Andrea Tubaro, Egidio Moja

**Affiliations:** 1 Department of Urology, Ospedale Sant’Andrea, “Sapienza” University of Rome, Rome, Italy; 2 Unit of Clinical Psychology, Department of Health Sciences, University of Milan, San Paolo Hospital, Milan, Italy; 3 CURA Centre, University of Milan, Milan, Italy

**Keywords:** Prostate, BPO, LUTS, Centred care, Treatment

## Abstract

**Background:**

Even though evidence based medicine, guidelines and algorithms still represent the pillars of the management of chronic diseases (i.e: hypertension, diabetes mellitus), a patient centred approach has been recently proposed as a successful strategy, in particular to improve drug adherence. Aim of the present review is to evaluate the unmet needs in LUTS/BPH management and the possible impact of a patient centered approach in this setting.

**Methods:**

A National Center for Biotechnology Information (NCBI) PubMed search for relevant articles published from January 2000 until December 2016 was performed by combining the following MESH terms: patients centred medicine, patient centered care, person centered care, patient centered outcomes, value based care, shared decision making, male, Lower Urinary Tract Symptoms, Benign Prostatic Hyperplasia, treatment. We followed the Preferred Reporting Items for Systematic Review and Meta-Analysis (PRISMA). All studies reporting on patient centred approach, shared decision making and evidence-based medicine were included in the review. All original article, reviews, letters, congress abstracts, and editorials comments were included in the review. Studies reporting single case reports, experimental studies on animal models and studies not in English were not included in the review.

**Results:**

Overall 751 abstracts were reviewed, out of them 87 full texts were analysed resulting in 36 papers included. The evidence summarised in this systematic review confirmed how a patient centred visit may improve patient’s adherence to medication. Although a patient centred model has been rarely used in urology, management of Low Urinary Tract Symptoms (LUTS) and Benign Prostatic Obstruction (BPO) may represent the perfect ground to experiment and improve this approach. Notwithstanding all the innovations in LUTS/BPO medical treatment, the real life picture is far from ideal.

**Conclusions:**

Recent evidence shows a dramatical low drug adherence and satisfaction to medical treatment in LUTS/BPH patients. A patient centred approach may improve drug adherence and some unmet needs in this area, potentially reducing complications and costs. However further well designed studies are needed to confirm this data.

## Background

In the last decades Evidence Based Medicine (EBM) has been the cornerstone of the clinical practice [1, 2]. Physicians’ personal experience and expertise are often limited by several knowledge biases and gaps, thus EBM intends to ameliorate the decision-making process by collecting and summarising evidence from well-designed and well-conducted clinical trials, developing and updating international, widely-accepted guidelines [1, 2]. Following this approach, a safer, more reliable, and more cost-effective clinical practice may be achieved.

Conversely, critics were concerned that the emphasis on EBM could undervalue the tacit knowledge that physicians may accumulate with clinical experience [1–3]. In addition they questioned whether results from designed research could apply strictly about real patients, who often differ significantly from those included in clinical trials [1–3]. Lastly EBM frequently ignores patients’ preferences and values, theoretically reducing their adherence to the proposed treatment [1–3].

Therefore, recently the patient centred approach has emerged as an important new paradigm in the clinical management of patients in many specialties including urology [1–3]. Impressive evidence supports positive associations between physician communication behaviours and positive patient outcomes, such as patient recall, patient understanding, and patient adherence to therapy [2]. Nonetheless, incorporating patient values, preferences and circumstances is probably the most difficult and important step in the management of urological diseases and frequently it does not receive the appropriate interest. As recently suggested by Hoffmann “Without shared decision making, EBM could turn into evidence tyranny” [4]. Lower urinary tract symptoms (LUTS) are a common complaint in adult men with a great impact on quality of life [5]. Medical treatment of LUTS due to Benign Prostatic Obstruction (BPO) represents the standard treatment aiming to improve symptoms, patient’s quality of life and reduce disease progression [6]. Despite EBM, algorithms and guidelines are the highway to guide LUTS/BPO treatment, outlooks are sometime far from reality and we know from daily practice that different medical needs remain unmet in this area. Therefore, the medical management of LUTS/BPO seems to be a fertile ground to experiment a patient centred approach. Probably LUTS/BPO patients may benefit from a shared decision method, aiming at discussing harms and benefits of different treatment options, taking into account personal expectations and personal feelings generated by the illness. LUTS/BPH management is based on evidence-based medicine although a patient centred approach could be proposed and integrated. Previous experiences in chronic diseases as diabetes and BPCO have confirmed that an integrated approach including evidenced based and patient centred medicine have a significant impact on patients care without contraindications.

**Aim of the present review is to evaluate the unmet needs in LUTS/BPH management and the possible impact of a patient centered approach in this setting.**

## Methods

**Fig. 1:**
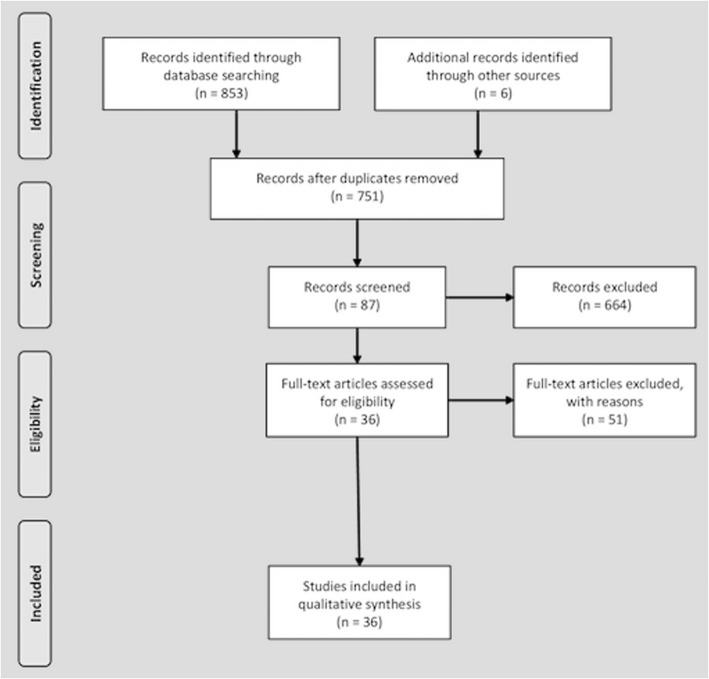
Flow diagram of the search results according PRISMA criteria

A National Center for Biotechnology Information (NCBI) PubMed search for relevant articles published from January 2000 until December 2016 was performed by combining the following MESH terms: patients centred medicine, patient centered care, person centered care, patient centered outcomes, value based care, shared decision making, male, Lower Urinary Tract Symptoms, Benign Prostatic Hyperplasia, treatment, drug adherence and measurements of adherence. We followed the Preferred Reporting Items for Systematic Review and Meta-Analysis (PRISMA). Only articles published in the English language and with an available full text were selected. In addition, sources in the reference sections of the identified publications were added to the list. Furthermore, all the abstracts presented at the annual congresses of the European Association of Urology (EAU) and American Urology Association (AUA), were evaluated and selected if relevant. All studies reporting on patient centred approach, shared decision making and evidence-based medicine were included in the review. All original article, reviews, letters, congress abstracts, and editorial comments were included in the review. Studies reporting single case reports, experimental studies on animal models, congress abstracts and studies not in English were not included in the review. The initial search resulted in 818 citations (Fig. 1). After initial title screening and manual reduplication, 749 references remained for abstract review. Four authors (CDN, FP, RL and EM) selected the initial studies based on selection criteria by abstract screening. These studies were categorised in three categories: excluded, included and possibly relevant. Included and possibly relevant studies were rescreened by three authors (CDN, FP and EM) to confirm eligibility. Overall 715 studies were excluded (not relevant to the topic or not original research). All authors then participated in full-text evaluation for the remaining 36 citations identified by abstract review or by manual search of references list (Fig. 1). Full texts were analysed by four reviewers (CDN, FP, RL, EM) and two subheadings were identified to summarize the results: LUTS/BPO medical treatment: unmet needs and Patient centered medicine in LUTS/BPO management (Table 1).’

**Table 1:** Characteristics of the studies retrieved

Study and year	Design	Main findings
ACCF 2012 [40]	Guidelines	Patient centered approach should be implemented in the management of the cardiological patient.
Agarwal 2014 [25]	Observational	Patient perception of urinary incontinence may differe from clinicians perception.
Balint 1969 [33]	Lecture	A shift of emphasis in the research from expecting the doctor to be a sort of detective inspector to a study of the varieties of response open to the doctor; or to put it in other words to the variety of ways the doctor can be used. This may be one of the changes which will lead to understand the possibilities and techniques of ‘patient-centred medicine’ and thus to undo the split in the doctor.
Bertaccini 2001 [29]	Observational	Quality of life is a major determinant in LUTS/BPH patients evaluated by the ICS-Qol questionnaire.
Cindolo 2014 [20]	Observational	Adherence to pharmacological therapy for BPH is low and could affect clinical outcomes. Our findings suggest the need for new strategies to increase patient adherence to prescribed treatment and more appropriate prescribing by physicians.
Cindolo 2015 [21]	Retrospective	Adherence to pharmacological therapy of BPH-associated LUTS is low and varies depending on drugs class. Patients under CT have a higher likelihood of discontinuing treatment for a number of reasons that should be better investigated. The study suggests that new strategies aiming to increase patient’s adherence to the prescribed treatment are necessary in order to prevent BPH progression.
Cornu 2010 [8]	Review	Major variations were seen among European countries concerning the prescriptions related to BPH, although the prevalence of the disease and the guidelines are similar. Analysis of actual prescription levels would complement evidence-based medicine as critical material for public health analysis, recommendations, and health insurance policies.
Coyne 2009 [31]	Observational	In this large population study of three countries, LUTS are highly prevalent among men and women aged > 40 years. In general, LUTS experienced ‘often’ or more are bothersome to most people.
Chung 2013 [27]	Prospective	LUTS are important risk factors in predicting the presence of clinically relevant depressive symptoms. In elderly men, increased awareness and possible screening are needed to detect the increased risk of clinically relevant depressive symptoms.
De Nunzio 2016 [7]	Review	The possibility of tailoring BPH treatment according to different patient characteristics and expectations, using two or more drugs, seems a promising path in the field of LUTS/BPH management; however, physicians should consider the risk of increasing costs without proven long-term efficacy with most of these combination treatments.
Epstein 2005 [24]	Rieview	PCC is regarded by the public, health care organizations, funding agencies and licensure bodies as a component of high-quality care. Defining outcomes of patient centeredness is essential to measure the clinical impact of a PCC approach.
Foo 2010 [9]	Review	The final decision for management of LUTS/BPH patient can then be tailored and individualized to achieve cost-effectiveness
Emberton 2007 [23]	Observational	This study highlights discrepancies between views and beliefs of patients and physicians regarding BPH and current practice in Europe.
Emberton 2010 [24]	Review	Improved physician–patient communication will help determine the best treatment option for patients with BPH and may ensure greater compliance and treatment success.
Foo 2017 [10]	Review	Treatment of prostatic adenoma can be individualized and tailored. Final decision-making would be personalized to the patient’s age, comorbidity and preferences (values). This would be in line with the recent emphasis on patient-centered care in evidence-balanced medicine, treating the patient not just the disease.
Fourcade 2008 [11]	Observational	There were geographical discrepancies that could be attributed to either different cultural habits or merely organisational differences, e.g. the presence of office urologists in Germany or diverse modes of access to phytotherapy (prescription vs ‘over the counter’) in the various countries.
Fourcade 2012 [12]	Observational	Around half of BPH patients medically treated report unsatisfactory outcomes, suggesting consequential unmet medical needs in general practice. A patient centered approach may improve outcomes.
Garraway 1993 [28]	Observational	Further investigation of these possible influences on non-consultation is required before any programme of health education can be considered which would encourage a higher proportion of men with bothersome urinary symptoms to come forward for attention at an earlier stage in the natural history of benign prostatic hyperplasia.
Greenhald 2014 [35]	Essay	Evidence based medicine has not resolved the problem sit set out to address (especially evidence biases and the hidden hand of vested interests),which have become subtler and harder to detect. Despite lip service to shared decision making, patients can be left confused and even tyrannised when their clinical management is inappropriately driven by algorithmic protocols,top-down directives and population targets.
Hong 2005 [5]	Review	Patient perceptions are receiving greater emphasis as part of clinical decision-making. Selecting an inappropriate treatment, or not including the patient’s preference, may lead to a cascade of therapies and unmet expectations, and increase the economic and human burden of the disease.
Hollingsworth 2009 [13]	Retrospective	On average, urologists had a higher intensity practice style for benign prostatic hyperplasia than primary care physicians. Further studies are needed to determine how these practice style differences relate to patient clinical outcomes.
Lamiani 2008 [37]	Prospective	Results suggest that the concept and practice of patient-centred care is variable and may be influenced by culture. The study methodology improved participants’ self-awareness of cultural values, and has potential as a cost-effective, experiential educational approach
Little 2001 [1]	Observational	Components of patients’perceptions can be measured reliably and predict different outcomes.If doctors don’t provide a positive,patient centred approach patients will be less satisfied,less enabled,and may have greater symptom burden and higher rates of referral.
Makoul 2001 [17]	Review	The group identified seven essential sets of communication tasks: (1) build the doctor-patient relationship; (2) open the discussion; (3) gather information; (4) understand the patient’s perspective; (5) share information; (6) reach agreement on problems and plans; and (7) provide closure. These broadly supported elements provide a useful framework for communication-oriented curricula and standards
Miner 2009 [15]	Review	General Practitioners and Urologist manage LUTS/BPH patients differently and not always according to the guidelines. Increasing communication between patients, GPs and Urologist may improve management of LUTS/BPH patients.
Mozes 1999 [26]	Observational	The relative weight of the impact of a symptom or disease on QoL domains is changed by the presence of other competingfactors, such as co-morbidities or sociodemographic attributes. Social context and quality of life is essential for a correct management of LUTS/BPH patients
Murray 2001 [3]	RCT	An interactive multimedia decision aid in the NHS would be popular with patients, reduce decisional conflict, and let patients play a more active part in decision making without increasing anxiety. The use of web based technology would reduce the cost of the intervention.
Piercy 1999 [46]	Observational	A shared decision making program is beneficial for the patient and should be implemented in clinical practice specially for LUTS/BPH patients. Patients were enthusiastic and physician-patient relationship could be enhanced.
Ridder 2015 [30]	Observational	The prevalence of LUTS, especially nocturia and urgency, is high and a significant number of men indicated to be seriously bothered. Increasing awareness of male LUTS, and storage symptoms in particular, is warranted to discuss management options that could increase quality of life.
Sells 2000 [32]	Prospective	The study confirmed the presence of significant morbidity in the partners of patients with BPE. The degree of partnermorbidity was related to the severity of the patients’ symptoms. Including the social entourage when managing LUTS/BPH patient may improve its management.
Stewart 2001 [34]	Editorial	Patients “may not prefer a patient centred approach” and hence its universal adoption would be “unwise.” Patient centred clinical practice is a holistic concept in which components interact and unite in a unique way in each patient doctor encounter.
Wei 2011 [14]	Observational	Significant differences in practice patterns were observed between primary care physicians and urologists in the evaluation of and management for lower urinary tract symptoms/benign prostatic hyperplasia. These data establish valuable benchmarks and identify possible interventions that may improve the standard of care.
Wagg 2012 [19]	Observational	Need for a better understanding of non-persistent patients treated with antimuscarinics and for the development of initiatives to improve the quality of drug therapy management. Further studies are required to investigate the reasons underlying this trend, such as lack of effi cacy, poor tolerability or inconvenient dosing, why patients are lost to follow-up, whether symptoms resolve at some point during the prescribed treatment, and whether lack of patient understanding about the need for long-term management is a factor.
Weston 2001 [22]	Comment	When you and your patient disagreeabout management, be sure to listen carefully to thepatient’s ideas and paraphrase them so that the patient knows that you understand his or her point of view. Then, express your concerns and engage in a discussion that seeks to find common ground.
WHO 2003 [16]	Review	Methods and interventions to improve drug adeherence.

## Results

Overall 36 articles were selected for the quantitative synthesis and divided in two topics: LUTS/BPO medical treatment: unmet needs and Patient centered medicine in LUTS/BPO management.

### LUTS/BPO medical treatment: unmet needs

LUTS can be divided into storage, voiding and post-micturition symptoms [6]. LUTS are highly prevalent, cause bother and impact on QoL. LUTS are strongly associated with ageing processes; therefore associated costs and burden are likely to increase with future demographic changes. Most elderly men suffer at least of one LUTS. LUTS evolve dynamically: for some patients LUTS persist and progress over long time periods, and for others they remit [6]. Six pharmacological classes [alpha blockers (ABs), 5-alpha reductase inhibitors (5ARIs), phytotherapeutics, antimuscarinics (AMs), beta-3 agonists and phosphodiesterase type 5 inhibitors] are available alone or in combination for the treatment of male LUTS [7]. However, notwithstanding all these different therapeutic options, medical treatment of LUTS/BPO is far from the ideal situation and different unmet needs remain in this area [8, 9].

Despite most of the European countries adopt the same guidelines for the management of patients with LUTS/BPO, different prescription strategies exist between European countries [10]. The overall prescription index is three times more important in southern countries than in northern countries [10]. In addition, when three classes of medications are compared, alpha-blockers are continuously widely used, 5-ARI prescriptions are variable (highest in Poland and Italy), and the prescription of plants is strictly country dependent (significantly higher in France and Hungary) [10].

Fourcarde et al., in a cross-sectional observational study, described the profile and management of patients receiving medical therapy for BPH in primary care-centres in France [11]. Half of the BPO patients medically treated report unsatisfactory outcomes and only 60% of patients received a stable treatment without modifications over a year time [11, 12]. Moreover, only 17.3% of the patients start with combination treatment and curiously the most prescribed combination therapy was alpha blockers + plant extract (49.7%), non-considered as a recommended combination treatment in the current guidelines, highlighting that only a small number of physicians adhere to guidelines and algorithms [11, 12].

LUTS/BPO patients are managed by urologists and general practitioners (GPs) and some differences in drug prescription can be commonly observed. The BPH Registry and Patient Survey is a longitudinal, observational, disease registry cohort of patients enrolled from January 2004 to February 2005 in the United States [13]. It includes 402 urologist and primary care physician practices throughout the United States. Several differences in prescription patterns may be seen between urologist and GPs. GPs tend to prescribe more likely ABs (77,4% vs 58,4%) than 5ARIs (14% vs 6,3%), AB plus 5ARI combination therapy (22,7% vs 13,8%) or anticholinergic therapy (4,8% vs 2,5%) [26]. Nonetheless, the abovementioned results are confirmed by other experiences in European and Asian cohorts [14, 15]. Therefore this evidence emphasizes the low observance of international guidelines from Urologists and GPs, and prompts a better collaboration between GPs and Urologists.

Another important concern in the LUTS/BPO management is represented by the poor drug adherence. Adherence to medication is best defined by the extent to which patients take medications as prescribed by their health care providers [16]. Persistence is defined as the mean number of days that a patient remained on therapy. The non-adherence and the lack of persistence to a certain medication have been recognized as a public health problem [16]. The current guidelines offer multiple and different chronic drug regimens for the treatment of BPO/LUTS [17, 18]. However very little is known on the patient adherence to the LUTS/BPO medications. Wagg et al. investigated patterns of persistence with oral AMs drugs across different age groups in UK [19]. The mean persistence rate ranged between 77 and 157 days depending on AM type and age (older patients presented better persistence rates) [19]. The same issue has recently been addressed by Cindolo et al. in their study including 1,5 million patients under LUTS/BPO medications [20]. The number of patients who received prescriptions for at least 6 months was 97,407, decreasing to 61,298 (63%) at 10 months and 28,273 (29%) at 12 months (26%). The proportion of patients who continued the drugs up to 10 months was 70, 59, and 34% respectively for AB, 5ARI, and combination therapy, respectively [20]. These results confirmed by similar experiences [21] showed as medical treatment of LUTS/BPO is far from the ideal treatment and that several factors could influence the long-term efficacy in relation to the poor drug adherence and persistence observed in real life studies.

Several factors as race, insurance coverage, information technology, type of medication and prescription burden can influence drug adherence in LUTS/BPO patients, however recent evidence support that drug adherence is mostly influenced by patient’s perception of discomfort and inconvenience and patient’s expectations [19, 21].

Therefore, the clinicians should not limit their attention to the correct diagnosis and treatment of the disease (LUTS/BPO); they are required to provide a comprehensive assessment of the patient’s illness experience. Nevertheless a thorough exploration of the illness experience requires insight, tools, and practice. One helpful acronym, that could summarize the issues to be considered in a patient centered approach, is FIFE: Feelings, Ideas, Function, and Expectations [22].

#### **F**eelings: what emotions have your experiences given rise to?

The occurrence of lower urinary tract symptoms unsurprisingly generates in the minds of patients several feelings, especially fears, related to this illness condition. According data from Emberton and coworkers survey, the main concerns first experienced by symptomatic patients seeking healthcare consultation were the fear of cancer, disruption to sleep, discomfort and embarrassment [23, 24]. In particular almost one-third of patients (32%) mentioned a fear of cancer, as the reason for seeking medical assistance, and those with more severe symptoms, were more likely to harbour this underlying concern. Other common complaints triggered by LUTS onset are: frustration with symptoms (18%), impact of symptoms on work life (10% of patients), impact of symptoms on social activities (9%), affection of the relationships with people (5%) [23, 24]. Furthermore Agarwal and coworkers reported that urgency, nocturia and urinary incontinence are the most bothersome symptoms in the their study population [25]. Interestingly, 10 weeks delay occurs usually from symptoms’ onset to medical advice [23]. The main motivations that induce patients to defer consultation are the hope that the symptoms would go away or the belief that symptoms were an expected component of ageing [23].

#### **I**deas: what do you think is causing this?

Despite more than half of patients (56%) affirmed that they felt ‘fairly’ or ‘very’ well-informed about health issues related to prostate problems, the prevalent **I**dea (32%) about what causes their symptoms was once again a cancer [23, 24]. When the disease is recognised as benign condition, patients’ concerns usually shift to a fear of subsequent disease progression. In particular 57% of subjects were significantly worried about the possibility of acute urinary retention (AUR), and 67% about surgery, while 68% believed that the insertion of a catheter would have a worse impact on their quality of life (QoL) than surgery [23, 24].

#### **F**unction: how has this affected your work? Relationships? Hobbies? Self-care?

We should not underestimate the impact of LUTS on the quality of life since LUTS affect patients’ **F**unctions as do several chronic diseases such as epilepsy, asthma [5]. Using the SF-36 and EuroQoL questionnaires Hong and coworkers reported that increasing symptom severity was significantly associated with worsening physical role, social functioning, vitality, mental health and perception of general health [5]. Furthermore, in all domains except physical functioning, patients with BPO had a worse QoL than patients with epilepsy or chronic pulmonary disease [5]. Sameway, Mozes et al. [37] showed a remarkable negative impact of LUTS on the mental health domain of QoL, which was greater than other disease states such as pulmonary disease. Consistently, in an Asian cohort of patients Chung et al. reported that the presence of moderate-to-severe LUTS at baseline were significantly associated with a three times increased risk for being depressed at two-year follow-up (OR = 2.97; CI: 1.70–5.20) [27]. In addition, in a Scottish community-based survey, half of men with LUTS/BPO experienced limitations with at least one living activity (e.g. the ability to sleep, participate in outdoor sports or to travel), while this degree of interference was reached by only 3% of subjects in the same age group without LUTS/BPO [28]. In accordance with these findings, in a cohort of Italian patients with bothersome LUTS (IPSS more than 7), Bertaccini et al. found that 95% of subjects would not be completely happy to spend the rest of their life with their actual condition and that LUTS/BPO presence influences their life from ‘a little’ to ‘a lot’ in 79% of patients [29]. All these surveys on the quality of life are agreed that storage symptoms are the most bothersome ones. In fact in all these studies QoL are more positively associated with storage symptoms (frequency, urgency or nocturia) than voiding symptoms (weak stream, hesitancy, etc.) or objective parameters (urinary flow, prostate volume, etc.) [26–28]. These conclusions were recently further confirmed in a large cohort of 5890 Belgium men aged ≥ 40 years (mean age: 61.2 years) [30]. Nocturia (69.2%) and urgency (58.3%) were the most prevalent and bothersome symptoms. Both prevalence and bother of all LUTS increased with age. Additionally, 28.9% of men reported to be a little bothered by their LUTS condition in everyday life, while 11.9% were bothered a lot/very much (2.5% in age group 40–49 years increasing to 29.2% in those > 80 years) [30].

#### **E**xpectations: what are you hoping to leave here with?

When clinicians plan a possible BPO/LUTS treatment, they should bear in mind the patients’ **E**xpectations. In the PROBE survey Emberton and co-workers reported that more than half of all patients had discussed the topic of prostate-related surgery or AUR with their healthcare provider, and most of them reported that they were ‘fairly’ or ‘very’ concerned about developing these complications [23, 24]. Further analysis from the PROBE survey and the Kaplan survey study has provided a better understanding of preferences and satisfaction with BPH (Benign Prostatic Hyperplasia) treatments, suggesting that patient and physician expectations may not always coincide [23, 31]. In the PROBE study, patients considered that the ideal treatment option is a drug providing a 50% reduction in the risk of surgery and symptom relief even if after 6 months, while the worst treatment is drug providing relief from symptoms within 2 weeks but no reduction in the risk of surgery in the long term [23]. The Kaplan survey confirmed how most of BPH patients are interested about long-term effect of treatment and their beliefs are completely different when compared with physicians. Most of them supposed that patients were more interested about immediate symptom relief than with long-term effects [31].

Finally another important element to consider during the shared decision-making is the “whole person” and the social context. In the attempt to give relief to urinary problems, we should also consider the other personal areas that might be involved and affected by this decision. Medical LUTS/BPH medications have a moderate impact on sexual life and in particular incidence of sexual AEs with combination therapy may be as high as 30% [21, 22]. Nevertheless clinicians often underrate the patients’ concerns about their sexual life. Fourcarde et al. described the profile and management of patients receiving medical therapy for BPH in primary care in four European countries [24, 25]. Even in return for complete suppression of urinary problems, most patients (> 50%) declared they would not agree to continue the treatment if they had to experience sexual adverse events [12].

If the patients agree, family members and in particular the partners should always be involved in the decision-making, as they are the main actors in the social context that surrounds the patients.

In fact, partners play a specific role in patient’s life as well as in in LUTS/BPH treatment. Sells and co-workers evaluated 90 partners using a dedicated questionnaire to assess partners’ morbidity associated with BPH/LUTS management [32]. Almost all partners experienced some morbidity as a consequence of the patients’ condition, with the most common issues being sleep disturbance, fear of cancer and surgery and limitations in social life including sexual life [32].

In conclusion, during this process, the clinician should not focus exclusively on the disease, but he should consider the whole person and the social context that surrounds him. In particular, in the case of LUTS/BPH, the clinician should evaluate carefully the sexual functions of the patient, involving as much as possible the partner in the decision-making.

### Patient centered medicine in LUTS/BPO management

The term “patient-centred” has been first used in a paper by Enid Balint in 1969 to indicate that the ‘whole person’ has to be considered in the clinical consultations [33]. Medical world showed a delayed reaction to this term and concept but in the last 20 years, there was progressively widespread acceptance that a ‘patient-centred’ approach may be beneficial [1, 2], although, as Stewart wrote: “Patient-centredness… may be most commonly understood for what it is not – technology-centred, doctor-centred, hospital-centred, disease-centred” [34]. Probably the rapid diffusion of a patient-centred model in several fields of medicine in the last decade has been linked to the crisis of the “Evidence-Based Medicine” model. As highlighted even by some members of the Centre for Evidence-Based Medicine of University of Oxford, evidence based medicine has not resolved all the problems it set out to address, which have become subtler and harder to detect [35]. In fact, patients often report that many of their informational and emotional needs remain unmet during encounters with their physicians and all this results in low levels of patient recall, a poor understanding of treatment recommendations, and a reduced adherence to those recommendations [35]. Therefore as suggested by the members of the Centre for EBM of University of Oxford, an exceeding of the standard EBM is needed, the research agenda should become broader and more interdisciplinary, embracing the experience of illness, the psychology of evidence interpretation, the negotiation and sharing of evidence by clinicians and patients [35]. Following these suggestions some authors have begun to explore the advantages of a patient centred approach in some chronic diseases, as hypertension, diabetes and arthritis [1, 2]. These studies showed that patients usually preferred patient centred care, and those who received it report enhanced health outcomes [1, 2]. In particular patient-centred encounters resulted in: better patient satisfaction, greater patient adherence to plans made, higher physician satisfaction, and fewer malpractice complaints [36].

Even if a patient-centred care has been interpreted and enacted differently among the different studies [37], it is possible to recognize two different dimensions of the concept. The first dimension means that in a patient-centred consultation the physician has to explore both the patients’ disease and four dimensions of the illness experience including: their feelings about being ill, their ideas about what is wrong with them, the impact of the problem on their daily functioning, and their expectations of what should be done [38]; the second dimension means that a patient-centred consultation has to encourage a more sharing, participative, and equal approach with the patient [38, 39]. The two dimensions are not mutually exclusive and affect the consultation and its outcomes. Therefore, the term “patient-centred” includes the patient perspective, and the psychosocial context along with shared understanding, power, and responsibility [40, 41]. In a recent consensus statement developed by representatives from medical education and professional organizations, seven essential communication tasks were identified: 1) build the doctor–patient relationship; 2) open the discussion; 3) gather information; 4) understand the patient’s perspective; 5) share information; 6) reach agreement on problems and plans; and 7) provide closure [17]. These tasks should be adopted in medical education, providing a template for the assessment of the various elements of patient-centred approach. Moreover the awareness of suboptimal health literacy and the importance of cultural competence in communication are imperative for effective patient communication and have been identified as key contributors to patient safety by the Joint Commission [18]. To reach significant enhancements of all these physicians’ communication skills, an effort by National Health Institutions is awaited, likely requiring changes in instruction at both the undergraduate and graduate levels of training [40]. Furthermore the challenge of assessing communication skills should not be underestimated [42]. This assessment should use well-established instruments for measurement of physicians’ communication skills in patient encounters [42].

In conclusion the patient-centred model has come a long way since the pioneering work of Enid Balint was published [33]. The scope of communication skills, important for successful clinical encounters, has been broadened and better defined. Moreover it has been successfully demonstrated that a patient-centred approach is positively associated with better health outcomes for patients in some fields of medicine [1, 2]. However, the potential of this method have not yet been explored in full, whereas in many areas of medicine, such as urology, the experiences with this approach although promising are still very rare. Further studies with this model are waited to confirm the positive outcomes of these preliminary findings.

A patient centered approach seems an innovative option to overcome the current limitations in the pharmacological treatment of LUTS/BPO patients, improving outcomes and drug adherence. As suggested in the last paragraph of the EAU guidelines on non-neurogenic LUTS, a patient centred care should be preferred and treatment should follow patients’ preferences and expectations in terms of efficacy, morbidity, speed of onset and disease progression [43]. Similarly the first paragraph of the NICE guidelines about the treatment of LUTS highlights a better communication between physicians and patients is mandatory, possibly using different communication skills and instruments in relation to patients’ education and needs [44]. Unfortunately, despite all these considerations, in the field of urology the use of this approach is still very limited. In particular, a total of seven survey studies, widely mentioned in the previous paragraph, have assessed LUTS/BPO treatment preference [11, 12]. Of these, two studies evaluated preferences in patients [11, 12], three studies examined preferences in physicians [13–15] and two studies investigated preferences in both patients and physicians [23, 24]. However, to our knowledge only two studies specifically addressed the impact of a shared-decision making approach for LUTS/BPO treatment outcomes [45, 46]. A preliminary RCT was performed in UK in the early 2000s to evaluate whether a decision aid on benign prostatic enlargement influenced patient decision-making, health outcomes, and resource use [45]. This study involved 33 GPs and 99 BPO patients, the decision aid consisted in interactive multimedia programme with booklet and printed summary [45]. Information included probabilities of the risks and benefits of each treatment, estimated on the basis of information on age, severity of symptoms, and general health entered by the patient at the beginning of the session. The final outcomes were promising, in fact the decision aid was highly acceptable to both the patients and their GPs; the decisional conflict was reduced in the intervention group and patients who accessed to the decision aid reported a more active part in the decision making process and were less anxious than control patients [45]. The study failed to reduce the rate of BPH surgical procedure, however the small sample size and the short follow up (9 months) may explain this inconclusive resul [45]. In the second study, including 678 patients with symptomatic BPH from eight Canadian centers, Piercy et al. examined the impact of a shared decision-making program (SDP) on perceived knowledge and treatment preference [46]. The SDP required by this study protocol, was rudimentary, consisting simply in viewing an educational programme designed to inform LUTS/BPO patients about their condition and treatment options [46]. SDP showed only a minor impact in changing the preferences of those subjects who had expressed an initial preference (89.7 and 89.4% of patients preferring surgical and non-surgical therapy respectively, maintaining their preferences after viewing the programme), although it helped almost half of those initially undecided in forming a preference, reducing the percentage of doubtful patients to 14.8% [46].

**Fig. 2:**
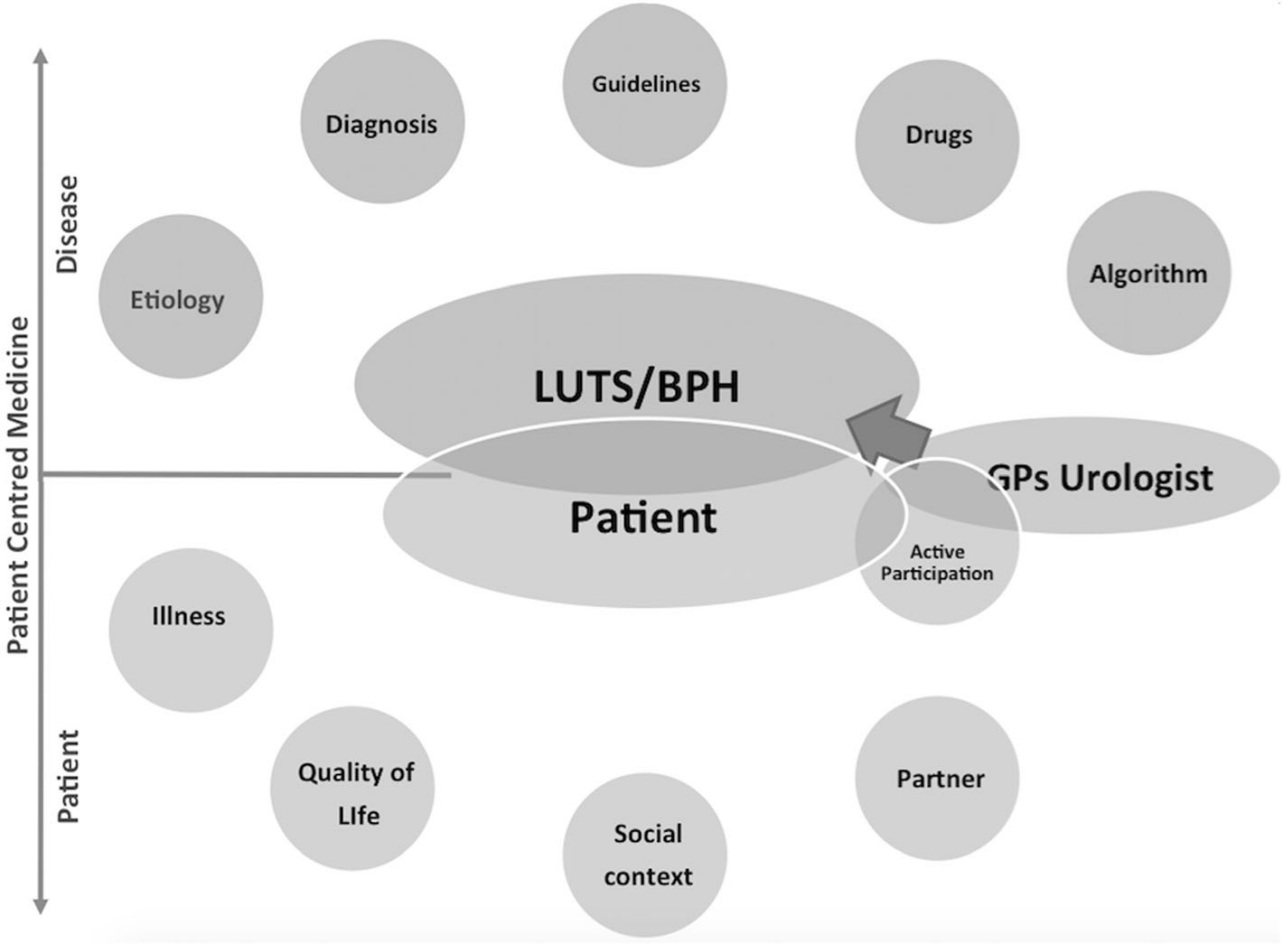
On the top, the traditional model of LUTS/BPH management and treatment, centred on disease’s characteristics and based on international guidelines and algorithms, derived by an Evidence Based Medicine approach. On the bottom, we suggest an implementation of the traditional model, through a strong collaboration between GPs and Urologists and a patient-centred model, with active participation of the patient during diagnostic evaluation and decision-making process. This novel model takes in account not only the disease’s characteristics but also the psychological dimensions of the illness experience

Figure 2 shows a possible suggestion of a patient centred approach in LUTS/BPH patients. Evidence based medicine includes etiology, diagnosis, current medications and guidelines/algorithms which should be integrated with patient illness, social context and partner. All the actors of a clinical consultation (GPs, urologist and patients) should participate together and actively communicate to achieve an integrated evidence based/patient centered approach. The disease and the patient are to be treated as a whole.

## Discussion

The present review analyses the possible impact of a patient centred approach in LUTS/BPH patients. The available literature on patient centred medicine has successfully demonstrated that this approach is positively associated with better health outcomes for patients in some fields of medicine. Medical treatment of LUTS/BPO is far from ideal, several factors could influence the long-term efficacy in relation to the poor drug adherence and persistence observed in real life studies. When managing LUTS/BPO patients, diagnostic and treatment algorithms should consider feelings, ideas, functions and expectations of the patient to tailor the management. Few studies evaluated the impact of a shared-decision making approach in the management of LUTS/BPO patients. Preliminary findings appear to be encouraging, even if definitive conclusions cannot be drawn from the scarcity of the available data. So far, our analysis should be considered as a preliminary summary of the role of a patient centred approach in managing patients with LUTS/BPO.

We strongly believe that LUTS/BPO is a particularly fertile ground for the implementation of a patient-centred approach. First, the plethora of guidelines and evidence-based therapies constitute a solid foundation from which evidence can be extracted and shared with patients [6, 7, 44]. Furthermore, several validated risk models of outcomes exist and can be used to advise patients of their likely outcomes, based on the results of previously treated patients [6, 7, 44]. Finally, there are many treatments for which no differences in outcomes are well defined; consequently the treatment plan may depend on patients’ feelings and expectations being then the appropriate driving force in decision-making.

Further studies on this topic are needed to confirm this hypothesis, however the experiences, derived from other specialties ahead of ours in this field [40, 47–49], suggest that in the next future the art of medicine probably will move more and more from a “one fit all” model to a “tailored” model, specifically for each patient’s needs. We support that in the near future Urologist and GP will enter a patient’s centered path for the management of LUTS/BPO where patient’s expectations, illness, ideas, social and familiar context were integrated with the disease and its relative possible treatment. The adoption of a patient centered model should improve patient’s care, the key is on focusing our attention rather than on the disease, on the patient who have the disease.

Additional studies should evaluate if a patient centered model, in a BPH/LUTS patient, can improve drug adherence, reduce the risk of doctor shopping and the number of legal controversies as already observed in other chronic diseases.

The management of the disease is then driven by the evidence based medicine however Feelings, Ideas, Function, Expectations, Social Context and partner take part into the decision making process where participation of each component of the central core represents the key for the successful treatment of the patient (Fig. 2). The next step will be how to translate effectively this theoretical model in a clinical scenario. The possible types of intervention could range between educational meetings, distribution of educational materials, audit and feedback, barriers assessment, and educational outreach visits and they could be divided in three categories of implementation intervention: 1) interventions targeting patients, 2) interventions targeting healthcare professionals, and 3) interventions targeting both. Anyway, regardless of the selected practical interventions to increase the SDP, the assessments of their efficacy should be addressed in terms of both patients’ satisfaction and clinical outcomes.

We have to acknowledge some limitations in our study. The few of studies retrieved on patient centred medicine in Urology do not allow definitive conclusion. Moreover, studies available on the subject have no common outcomes and therefore a quantitative analysis is limited. As well the studies available on patient centred medicine in other medical areas have different definitions of patient centred approach and analyse different aspects of the topic [40]. Probably, common outcomes are needed to better understand the real impact of a patient centred approach in the every day clinical practice. In particular, drug adherence in LUTS/BPH patients could serve as a proxy to evaluate the impact of a patient centred model. Notwithstanding all these limitations a patient centred approach may help clinicians in the management of LUTS/BPH patients and standing to the available evidence no real complications seem to emerge from this approach.

## Conclusion

LUTS/BPO medical treatment is a successful story in the field of Urology and it is based on excellent evidence and several International guidelines. However recent evidence shows a dramatically low drug adherence and satisfaction coming from LUTS/BPO patients on medical treatment. Urologists and general practitioners should be aware that a patient centred approach could improve drug adherence and some unmet needs in this area, potentially reducing complications and costs. Medical treatment should be considered in relation to patients’ illness, preference and expectations. The adoption of a patient-centered model in other chronic pathologies, such as diabetes and hypertension, have further improved drug adherence, patients’ compliance to a chronic treatment and have reduced a doctor shopping.

Similarly LUTS/BPH management may represent the perfect ground to experiment and improve this approach, considering the richness of the agenda’s components in these patients, the low drug-adherence rate reported in the literature, and the choice between several therapies of similar efficacy but with different effects on the patient’s QoL (with the importance to strongly involve every single patients in the treatment decision). However, the benefits of this approach, albeit reasonably deducible, are difficult to demonstrate in accordance with the criteria of the evidence base medicine and the adoption of a shared decision making is still very limited in the field of urology. The selection criteria in the medical treatment of LUTS remain primarily, as emerged from the aforementioned surveys, the personal preferences of the clinicians and the habit of prescribing a certain class of medication and this may explain the geographical spread above reported.

We proposed and support a patient centered model to improve drug adherence and some unmet needs in this area, potentially reducing complications and costs. Further studies in this area are awaited to support this hypothesis.

## Abbreviations

5ARIs: 5-alpha reductase inhibitors
ABs: Alpha blockers
AMs: Antimuscarinics
AUA: American Urology Association
AUR: Acute urinary retention
BPH: Benign Prostatic Hyperplasia
BPO: Benign Prostatic Obstruction
EAU: European Association of Urology
EBM: Evidence Based Medicine
LUTS: Low Urinary Tract Symptoms
PRISMA: Preferred Reporting Items for Systematic Review and Meta-Analysis
SDP: Shared decision-making program
